# 2D Material Optoelectronics for Information Functional Device Applications: Status and Challenges

**DOI:** 10.1002/advs.202000058

**Published:** 2020-04-08

**Authors:** Teng Tan, Xiantao Jiang, Cong Wang, Baicheng Yao, Han Zhang

**Affiliations:** ^1^ Key Laboratory of Optical Fiber Sensing and Communications (Education Ministry of China) School of Information and Communication Engineering University of Electronic Science and Technology of China Chengdu 611731 China; ^2^ Shenzhen Key Laboratory of Micro‐Nano Photonic Information Technology Guangdong Laboratory of Artificial Intelligence and Digital Economy (SZ) International Collaboration Laboratory of 2D Materials for Optoelectronic Science and Technology College of Physics and Optoelectronic Engineering Shenzhen University Shenzhen 518060 China

**Keywords:** 2D materials, information devices, nonlinear optics, optoelectronics

## Abstract

Graphene and the following derivative 2D materials have been demonstrated to exhibit rich distinct optoelectronic properties, such as broadband optical response, strong and tunable light–mater interactions, and fast relaxations in the flexible nanoscale. Combining with optical platforms like fibers, waveguides, grating, and resonators, these materials has spurred a variety of active and passive applications recently. Herein, the optical and electrical properties of graphene, transition metal dichalcogenides, black phosphorus, MXene, and their derivative van der Waals heterostructures are comprehensively reviewed, followed by the design and fabrication of these 2D material‐based optical structures in implementation. Next, distinct devices, ranging from lasers to light emitters, frequency convertors, modulators, detectors, plasmonic generators, and sensors, are introduced. Finally, the state‐of‐art investigation progress of 2D material‐based optoelectronics offers a promising way to realize new conceptual and high‐performance applications for information science and nanotechnology. The outlook on the development trends and important research directions are also put forward.

## Graphical abstract

Herein, the latest research progress and development trends of optoelectronic information devices based on 2D materials are summarized. This work gives a comprehensive review of this field, covering the optoelectronic properties of 2D materials, preparation and composite processes, as well as specific applications and prospects, aiming to provide readers a communication platform with references.

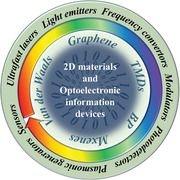

## 1 Introduction

A 2D material is a crystal in the form of a planar structure volume, which can be regarded as a 3D crystal with a negligible thickness in one dimension. The thickness of mono‐ or few‐atomic layer 2D materials is on the orders of magnitude smaller than the wavelength of light involved. Graphene, a material of monoatomic layer, which was successfully isolated by Geim's group in 2004,^[^ ^1^ ^]^ opens the playground of 2D materials and enables researchers to explore the special properties of materials in unprecedented vision. In the past decade, in addition to graphene, more and more 2D materials have been successfully introduced, including transition metal dichalcogenides (TMDs),^[^ ^2^ ^]^ black phosphorus (BP),^[^ ^3^ ^]^ MXenes, and their van der Waals heterostructures (vdWs).^[^ ^4^ ^]^ Up to date, the 2D material research system is increasingly perfect with the efforts of many researchers. The combination of 2D materials and optics has become a mainstream trend, attracting increasing attention for applications in electronics, photonics, and optoelectronics.^[^ ^5^ ^]^

2D materials, fundamentally different from their bulk parents, exhibit a rich variety of physical properties thanks to their diverse electronic structures, ranging from large band insulators to narrow gap semiconductors, topological insulators, semimetals, and metals. The highly tunable bandgap offers an extremely wide range of optical responses.^[^ ^6^ ^]^ Furthermore, they are easy to integrate with photonic structures such as fibers^[^ ^7^ ^]^ and chips,^[^ ^8^, ^9^ ^]^ because their surfaces are naturally passivated without any dangling bonds. Finally, most 2D materials have excellent electrically tunable physical properties, so they are very suitable for functionalized photoelectric information devices.

To date, many experts in related fields have reviewed the application of 2D materials in optoelectronics.^[^ ^10^, ^11^, ^12^, ^13^, ^14^, ^15^ ^]^ However, the optoelectronic information devices based on 2D materials is undoubtedly an important topic in the field of information science, yet have not been comprehensively reviewed. Herein, the optical and photoelectric properties of various 2D materials are briefly introduced at first. Then, the commonly used 2D material preparation processes, including mechanical exfoliation (ME), liquid phase exfoliation (LPE), chemical vapor deposition (CVD), and van der Waals composite preparation process, and the typical composite optical structures are analyzed and compared. After that, the up‐to‐date optoelectronic information devices based on 2D materials, including ultrafast lasers, light emitters, frequency convertors, modulators, detectors, plasmonic generators, and sensors, are reviewed. Finally, our prospects on the future perspectives of 2D layered materials and their applications are presented.

## 2 Photonics and Optoelectronics of 2D Materials

**Figure 1:**
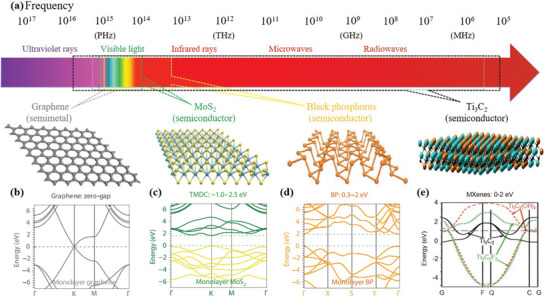
Ultra‐broadband photoresponse of 2D materials from UV to microwave regime. a) Electromagnetic spectrum and the atomic structures of graphene, MoS_2_, and BP are shown in the bottom of the panel, left to right. The possible spectral ranges covered by different materials are indicated using colored polygons. Band structures of b) single‐layer graphene, c) MoS_2_, and d) BP. Reproduced with permission.^[^ ^6^ ^]^ Copyright 2014, Nature Publishing Group. Reproduced with permission.^[^ ^35^ ^]^ Copyright 2017, Springer Nature. e) Band structure of MXene monolayer with —OH and —F surface termination and no termination (Ti_3_C_2_). Reproduced with permission.^[^ ^34^ ^]^ Copyright 2011, Wiley‐VCH.

The research on 2D materials was initially triggered by the groundbreaking work on graphene.^[^ ^1^ ^]^ Since then, graphene and other 2D materials have been widely studied on their unique and fascinating electrical, optical, mechanical, thermal, and chemical properties. So far, a number of 2D materials have been fabricated and many remarkable optical properties have been discovered (e.g., ultrafast broadband optical response ranging from ultraviolet to radiowaves, strong and tunable light–mater interaction, and large optical nonlinearities), because of the diverse electronic properties and bandgap structures as shown in **Figure** **1**. In this section, we mainly focus on the photonic and optoelectronic properties of 2D materials, including graphene, TMDs, BP, MXenes, and van der Waals heterostructures.

### 2.1 Graphene

**Figure 2:**
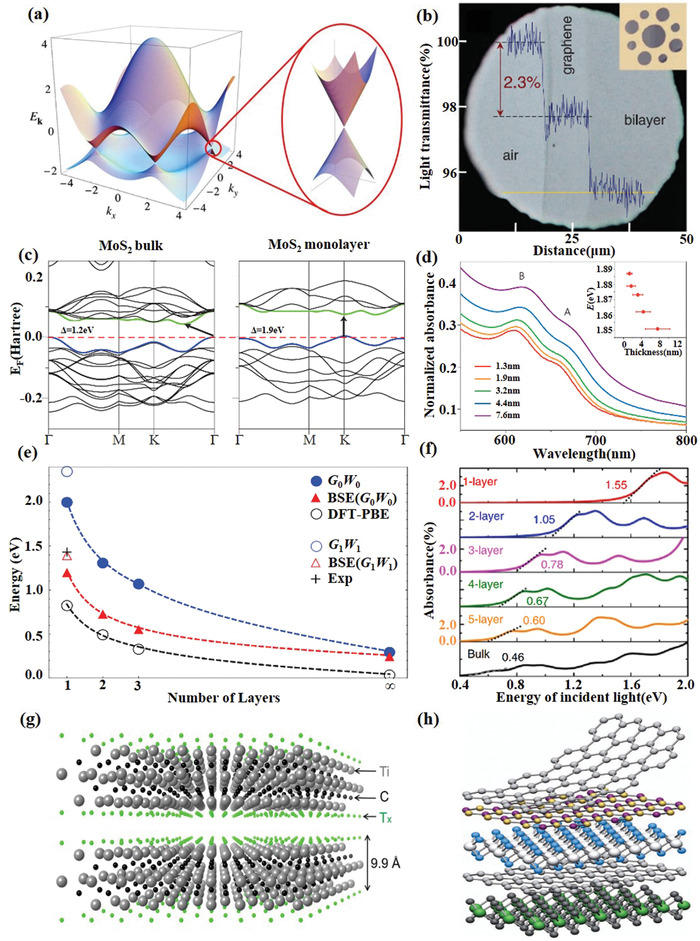
Characterizations of types of 2D materials. a) Electronic band structure of graphene. Reproduced with permission.^[^ ^16^ ^]^ Copyright 2009, American Physical Society. b) Light absorption of graphene monolayer. Reproduced with permission.^[^ ^18^ ^]^ Copyright 2008, American Association for the Advancement of Science. c) The band structure of bulk and monolayer MoS_2_. d) Absorption spectra of MoS_2_ thin films with average thicknesses ranging from 1.3 to 7.6 nm. Reproduced with permission.^[^ ^22^ ^]^ Copyright 2012, Nature Publishing Group. e) The evolution of bandgap calculated by different methods and optical absorption peak according to the stacking layer number of few‐layer phosphorene. Reproduced with permission.^[^ ^25^ ^]^ Copyright 2014, American Physical Society. f) Optical absorption spectra of few‐layer BP for light incident. Reproduced with permission.^[^ ^32^ ^]^ Copyright 2014, Nature Publishing Group. g) Crystal structure of typical MXene Ti_3_C_2_T*_x_*. h) van der Waals heterostructure. Reproduced with permission.^[^ ^4^ ^]^ Copyright 2013, Nature Publishing Group.

Graphene, the first member of 2D family, offers several advantages compared with other 2D materials due to its Dirac‐core‐like gapless energy structure where the valence band meets the conduction band at the Fermi level,^[^ ^16^ ^]^ as shown in **Figure** **2**a. Therefore, graphene can interact with light from ultraviolet to the far‐infrared, and even to the terahertz and microwave regions (Figure 1a) due to its unique linear energy–momentum dispersion relation.^[^ ^17^ ^]^ And simultaneously, graphene exhibits strong light‐material interactions that a single layer of graphene absorbs 2.3% of the vertically incident light in the visible and near infrared spectrum, with absorption coefficient precisely defined by *πα*, where α = *e* ^2^/*ℏc* denotes the fine‐structure constant (Figure 2b).^[^ ^18^ ^]^ Noteworthy, the Fermi level of graphene can be tuned by electrical gating and chemical doping. This property allows us to precisely control graphene for infrared and visible light manipulation. Furthermore, graphene exhibits ultrafast carrier dynamics, high carrier mobility,^[^ ^19^ ^]^ wavelength‐independent absorption, tunable optical properties through changing the Fermi levels,^[^ ^20^ ^]^ low dissipation rates and strong electromagnetic field confining ability. And graphene also exhibits strong nonlinearity associated with exceptionally high third‐order susceptibility within the visible and near‐infrared spectral range.^[^ ^21^ ^]^ Finally, graphene is chemically stable and mechanically robust because of the strong covalent bonding between carbon atoms. All these properties of graphene make itself of great application value in broadband tunable devices especially in the far‐infrared and terahertz regions.

### 2.2 Transition Metal Dichalcogenides

Transition metal dichalcogenides (TMDs), the most studied member of the 2D material family beyond graphene, are atomically thin semiconductors of the type MX_2_ (M = Mo, W, Re; X = S, Se, Te). Since monolayer TMDs are typically semiconductor materials, their optical properties are different from those of graphene significantly, offering a broader and complementary application field. Their bandgaps cover the energy range from 1 to 2.5 eV, corresponding to the spectral range from near‐infrared to the visible (Figure 1c). One of the significant properties of the TMDs is the indirect‐to‐direct bandgap transition that occurs when the material thickness is reduced from multilayer to monolayer. For instance, MoS_2_, MoSe_2_, WS_2_, and WSe_2_ are indirect bandgap semiconductors in bulk form, but at monolayer their bandgaps transfer to direct ones (Figure 2c).^[^ ^22^ ^]^ Additionally, TMDs exhibit unusually strong light–material interactions that monolayer TMDs materials can even absorb 20% of the light at a specific resonance energy.^[^ ^23^ ^]^ Moreover, odd‐layer TMDs allows for second‐order and other even‐order nonlinearities that do not exist in graphene and even‐layer TMDs, which is attributed to the inverse symmetry broken. These properties are advantageous for applications requiring strong light absorption, such as mode locking lasers, and nonlinear optical device applications,^[^ ^24^ ^]^ such as second harmonic generation.

### 2.3 Black Phosphorus

Similar to TMDs, black phosphorus (BP), another direct bandgap semiconductor, can modulate the bandgap through changing the number of layers (Figure 2e). This layered material with a moderate bandgap of 0.3 eV in its thin‐film form. The bandgap of BP is expected to increase monotonically as the number of layers decreases that is widely tunable to around 2 eV in its single‐layer form known as phosphorene (Figure 1d). It bridges zero‐bandgap graphene and relatively wide bandgap transition metal dichalcogenides^[^ ^25^ ^]^ and covers a wide range of wavelengths from the mid‐infrared to the visible spectrum for light detection,^[^ ^26^ ^]^ modulation,^[^ ^27^, ^28^, ^29^ ^]^ generation applications and even biophotonics.^[^ ^30^, ^31^ ^]^ However, unlike the centrosymmetric of graphene and TMDs, the in‐plane conductivity and optical conductivity of black phosphorus are anisotropic due to the puckered structure, which leads to the high anisotropy of the light absorption and photoluminescence of black phosphorus.^[^ ^32^, ^33^ ^]^ Finally, it is worth mentioning that the electronic and optical properties of BP degrade rapidly, because BP lacks stability in the air. So, using BP for practical applications remains a challenge.

### 2.4 MXenes

In recently years, MXenes as a novel 2D material family member, has attracted significant attention from varies research communities since the first debut in 2011.^[^ ^34^ ^]^ MXenes represent 2D transition metal carbides, nitrides, and carbonitrides, and have a general formula of M*_n_*_+1_X*_n_*T*_x_*, where M is an early transition metal, X is C and/or N, T is surface terminations, and *n* = 1, 2, or 3. The first MXene Ti_3_C_2_T*_x_* was isolated via selectively extract Al from its MAX parent Ti_3_AlC_2_ in aqueous HF solution. Since then, the aqueous acid etching method has been widely employed to synthesis new MXene materials, such as Ti_2_C, Ti_3_CN, Ta_4_C_3_, V_2_C, Nb_2_C, and so forth.^[^ ^35^ ^]^

Compared to graphene, the easier of surface decorations may offers MXene a broader application vision in the chemical and biological area. However, the surface terminations might also hinder the exploration of basic physic properties of printing MXene. Recently, chemical vapor deposition (CVD) method has successfully applied to synthesis large size atomically thin MXene layer, which opens up the window for the investigation of more basic physics in MXene without surface terminations.^[^ ^36^ ^]^

Typically, nonterminated MXenes are metallic with a high density of states (DOS) at the Fermi surface due to the out layer of transition metal elements. The passivation of the surface might draw the DOS to a lower level, leading to the metal to semiconductor, insulator or semimetal transfer of MXene (Figure 1e).^[^ ^34^ ^]^ The reported lowest work function was predicted in methoxylationized niobium carbide Nb_3_C_2_(OCH_3_)_2_ MXene with a value of 0.9 eV.^[^ ^37^ ^]^ The ultralow work function of MXenes is suggested to be promising as field emitter cathodes and thermionic devices. A low optical attenuation (2–3% per nanometer) of MXene has been experimentally observed on varies substrates.^[^ ^38^ ^]^ This value is comparable the well‐known graphene (2.3% for monolayer). Considering the high conductivity of ≈10 000 S cm^−1^,^[^ ^39^ ^]^ MXene is suggested to be a promising transparent conductive material. An excellent broadband nonlinear optical responses of MXene have recently been demonstrated, which offers the opportunity for ultrafast photonics, plasmonic as well as all‐light modulations.^[^ ^40^, ^41^, ^42^ ^]^

### 2.5 van der Waals Heterostructures

Atomically thin 2D materials with wide‐ranging properties can be manufactured and engineered independently and then stacked together to form van der Waals‐bonded heterostructures, as shown in Figure 2h, offering surprising new opportunities for functional devices by combining different materials.^[^ ^43^, ^44^ ^]^ There have been tremendous efforts to explore different 2D heterostructures, including but not limited to graphene‐hexagonal boron nitride (hBN), graphene‐BP, TMD‐hBN, graphene‐TMDs, and TMD–TMD heterostructures. The free combination of different materials via the van der Waals heterostructure may lead to more exciting discoveries of novel electronic and optical properties. For example, Under van der Waals forces, different atomically thin 2D materials can be easily vertically stacked together to make 2D heterostructures without the conventional “lattice mismatch” issue, offering a flexible and easy approach to design desired physical or optical properties.^[^ ^4^ ^]^ At the moment, van der Waals heterostructures reveals unusual properties and new phenomena and shows great potential in functional optoelectronic devices, but it is still at an exploratory stage.

## 3 Device Fabrication and Applications

### 3.1 Synthesis Methods for 2D Materials

With the in‐depth study of 2D materials, a variety of methods have been developed to synthesize 2D materials, mainly including mechanical exfoliation, liquid phase exfoliation, chemical vapor deposition, and van der Waals epitaxial growth. Mechanical exfoliation method, commonly known as the scotch‐tape method which is popular for lab‐based studies, is a traditional technique to obtain atomically and few‐layer 2D materials,^[^ ^1^ ^]^ which has the advantages of low cost and easy operation with the trade‐off low yields and lacks controllability in uniformity, size, and thickness of the peeled flakes. To increase the yields, liquid phase exfoliation method with low cost and large‐scale production capacity has been developed, but it features the drawback of poor uniformity of layer thickness, and low yields of large flakes and single layers. Compared to the above two methods, chemical vapor deposition method was widely used to synthesize large area homogeneous 2D materials with controllable thickness and excellent properties. Here, we briefly discuss the merits of different synthesis processes. The fabrications and characterization techniques of 2D materials are of crucial for successful device realizations. In the following, the merits of above‐mentioned synthesis techniques are briefly elaborated and discussed, specifically for the functional information devices, in addition to the previous reviews.^[^ ^45^, ^46^, ^47^, ^48^ ^]^ These techniques enable flexible implementation of 2D material‐based hybrid optical structures, such as fibers and chips.^[^ ^49^, ^50^ ^]^

### 3.2 Transfer Process

The wet transfer method and the dry transfer method are two main methods to realize the composite structure of 2D material and optical components. Here, we briefly describe the operation flow of the two methods, and analyze their advantages and disadvantages as well as the appropriate applications.

**Figure 3:**
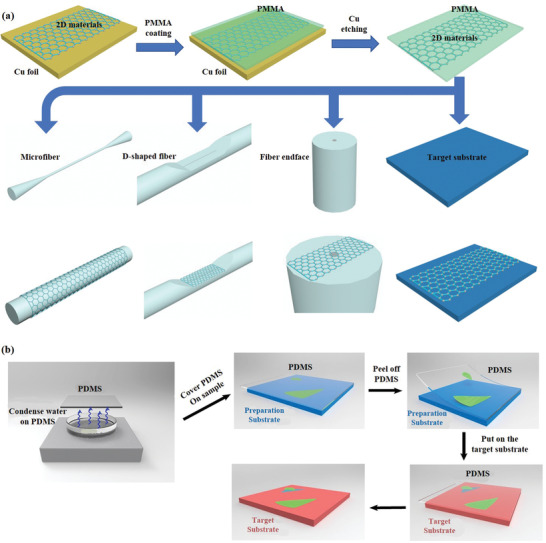
Conceptual diagram and fabrication of typical 2D materials based optoelectronic structures. a) Schematic diagram of the wet transfer technology. b) Schematic diagram of the capillary‐force‐assisted dry transfer procedure. Reproduced with permission.^[^ ^52^ ^]^ Copyright 2017, American Chemical Society.

The operation flow of wet transfer method: First, polymethyl methacrylate (PMMA) was spun on the upper surface of the 2D materials/copper composite layer, and then solidified PMMA. Second, the PMMA/2D materials/copper composite layer was placed in FeCl_3_ solution to dissolve copper layer by displacement. The PMMA/2D materials composite film with the copper layer removed floated on the surface of the solution. Then, PMMA/2D materials flexible films were soaked and cleaned in deionized water (DIW). Third, PMMA/2D materials composite film was combined with waveguide, as shown in **Figure** **3**a. The PMMA/2D materials/waveguide composite structure was then dried and shaped. Finally, the formed PMMA/2D materials/waveguide composite junction was constructed in acetone or acetone vapor to remove the PMMA layer.

The wet transfer technology is simple, easy to operate and has a high success rate, which is suitable for most of the current process requirements of 2D material‐based optoelectronic devices, especially waveguide structures (e.g., optical fibers) with curved surfaces. However, this method also has the following disadvantages: First, the surface tension generated by solution volatilization in the transfer process easily leads to the bending, stretching, and folding of the supporting layer, which leads to the folding and tearing of the 2D material film after the transfer. Second, PMMA residue is difficult to be completely removed and cleaned. The interface between the 2D material and the target substrate is contaminated with foreign substances and water molecules, which increases carrier scattering and affects device performance. Third, acetone is usually used to dissolve PMMA, limiting the use of partially soluble flexible polymer wood plaque substrates.

The operation flow of dry transfer method:^[^ ^51^ ^]^ First, the mechanical exfoliation 2D materials are pasted on the polydimethylsiloxane (PDMS) with good viscoelasticity, and the glass slide/PDMS/2D materials and target substrate are fixed on the nanopositioning systems and sample table, respectively. Second, the 2D materials was precisely shifted to the target position and lowered to contact the sample. Third, the 2D material film would stay on the target substrate since the adhesion between the 2D material and the target substrate was greater than that between the PDMS and the 2D materials, when glass slide/PDMS was raised. Due to the weak adhesion of PDMS in exfoliating 2D materials, the exfoliating process is difficult and the success rate of transfer is not high. Ma et al. developed a capillary‐force‐assisted clean‐stamp technique that uses a thin layer of evaporative liquid (e.g., water) as an instant glue to increase the adhesion energy between 2D crystals and PDMS for the pick‐up step. After the liquid evaporates, the adhesion energy decreases, and the 2D crystal can be easily released to the target substrate. The thin liquid layer is condensed to the PDMS surface from its vapor phase, which ensures the low contamination level on the 2D materials and largely remains their chemical and electrical properties,^[^ ^52^ ^]^ the detailed fabrications produces are illustrated in Figure 3b. This method is suitable for on‐chip transfer of materials such as graphene, TMDs and black phosphorus.

As a whole, compared to the wet transfer technology, this transfer method through physical adsorption does not introduce any chemical liquid in the transfer process, avoids chemical pollution, and better ensures the material surface clean and no residue after the transfer. Therefore, it is usually used in optical applications that require high surface cleanliness (e.g., microcavity). Meanwhile, the transfer position can be precisely controlled to realize vertical superposition of different materials such as insulator, semiconductor and metal, which is an efficient laboratory method for constructing high‐quality 2D heterojunction.

So far, the technology of transferring 2D materials to optical fibers and chips has been relatively mature, but some researchers still make efforts to improve the quality and success rate of transfer process.^[^ ^52^, ^53^, ^54^ ^]^

### 3.3 Composite Structures and Applications

**Figure 4:**
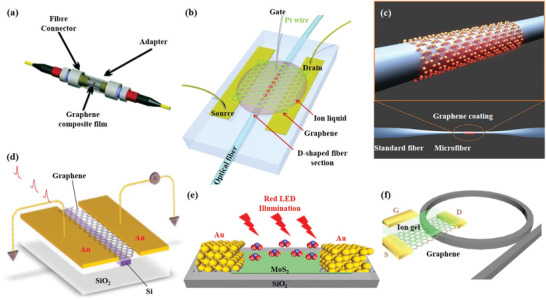
Applications of typical 2D materials based optoelectronic structures. a) The SA material is deposited on the end of the fiber for ultrafast lasers. Reproduced with permission.^[^ ^57^ ^]^ Copyright 2010, American Chemical Society. b) Graphene‐covered D‐shaped fiber for electro‐optic modulators. Reproduced with permission.^[^ ^115^ ^]^ Copyright 2015, Nature Publishing Group. c) Schematic of a graphene‐clad microfiber all‐optical nonlinear devices. Reproduced with permission.^[^ ^112^ ^]^ Copyright 2014, American Chemical Society. d) Integration of graphene and silicon waveguides for photodetectors. Reproduced with permission.^[^ ^133^ ^]^ Copyright 2013, Nature Publishing Group. e) Au/MoS_2_/Au‐based BJT was deposited on SiO_2_ substrates for gas sensors. Reproduced with permission.^[^ ^182^ ^]^ Copyright 2019, American Chemical Society. f) Schematic architecture of the graphene‐based microresonator. Reproduced with permission.^[^ ^9^ ^]^ Copyright 2018, Springer Nature.

Based on the design of optical structure and material characteristics, we classify 2D material‐based optoelectronic devices into two categories according to structure: 2D material‐based optical fiber devices and 2D material‐based on‐chip devices. Among them, typical optical fiber/2D materials composite structure include: 1) fiber end‐facet deposited 2D materials (**Figure** **4**a), 2) D‐shape fiber with 2D materials deposited on its top surface (Figure 4b), and 3) 2D materials wrapped microfiber (Figure 4c). Combined with optical fiber platforms, 2D materials offer unparalleled application advantages in the field of ultrafast lasers and sensing. Specifically, 2D material‐based ultrafast lasers are usually implemented by depositing 2D materials on the end‐facet of the optical fiber and utilizing its saturation absorption effect to form a saturated absorber, such as a single layer of graphene absorbs 2.3% of the vertically incident light. 2D materials wrapped microfiber and deposited on D‐shape fiber have longer light–matter interaction distance and interaction time than deposits on the fiber end‐facet. Therefore, these two structures are usually used in sensor design and rarely used in lasers, because they will bring a large insertion loss, but the long light–matter interaction distance and time will bring an extreme increase in sensor sensitivity. By changing the conductivity of 2D material through external parameters, the modulation of the optical field in the optical fiber can be realized.

Compared with the optical fiber platform, the on‐chip platform has higher requirements for the quality of 2D material and the transfer process, but has a high degree of integration. Currently, 2D material‐based on‐chip platforms, including waveguides (Figure 4d), SiO_2_ substrate (Figure 4e), and microrings (Figure 4f), have been widely used in optical communication systems (including lasers, modulators, detectors) and fundamental researches (including nonlinear optics, plasmon, and optical frequency combs). Most of these applications are based on the combination of 2D material‐based bipolar junction transistor (BJT) and field effect transistor (FET) and on‐chip platforms. It has great application potential in information science and functional optoelectronic devices.

## 4 Photonic Devices Using 2D Materials

### 4.1 Ultrafast Lasers

**Figure 5:**
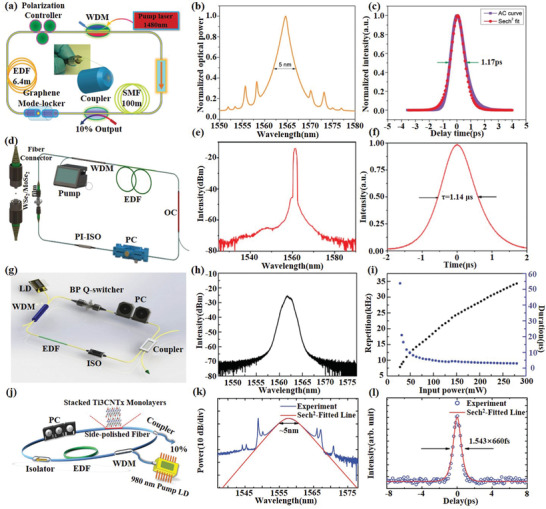
2D material‐based ultrafast lasers. a) Laser configuration constituting a ring cavity. b) Output pulse spectrum, centered at 1567 nm, with soliton sidebands. c) Autocorrelation trace of laser output and sech^2^ fitting curve. Reproduced with permission.^[^ ^7^ ^]^ Copyright 2009, Wiley‐VCH. d) Schematic diagram of the passively Q‐switched pulse fiber laser on the basis of WSe_2_ and MoSe_2_ SAs. e) Optical spectrum of passively Q‐switched pulse fiber laser based on the WSe_2_ SAs. f) Single pulse sequence at maximum pump power of 680 mW. Reproduced with permission.^[^ ^68^ ^]^ Copyright 2018, OSA Publishing. g) Schematic illustration of the ring cavity of the Q‐switched fiber laser based on BP SAs. h) Optical spectrum of the Q‐switched fiber laser based on BP SAs. i) Pulse repetition rate and duration versus incident pump power. Reproduced with permission.^[^ ^72^ ^]^ Copyright 2015, Wiley‐VCH. j) The schematic of the ring‐cavity erbium‐doped fiber laser incorporating the stacked Ti_3_CNT*_x_* SAs. k) Optical spectrum of laser output and sech^2^ fitting curve. l) Autocorrelation trace of laser output and sech^2^ fitting curve. Reproduced with permission.^[^ ^78^ ^]^ Copyright 2017, Wiley‐VCH.

Ultrafast lasers have been widely studied as important components of current optical communication systems. Currently, the commercial ultrafast lasers are realized by using semiconductor saturable absorber mirrors (SESAMs) as saturated absorbers. Its working bandwidth is limited, because the commercial SESAMs are based on resonant nonlinearity. Besides, high manufacturing costs also limit the further applications, which forced us to look for new saturated absorbing materials with ultrawide operating bandwidth and universality. In recent years, 2D materials have shown great potential in pulse laser generation due to their unique optical properties, such as broadband absorption, short recovery time, low saturation fluence, and high modulation depth. They have been used as saturable absorbers (SAs) for different lasers (solid‐states, fibers, semiconductors) operating at different wavelengths ranging from visible to MIR (500–2500 nm).^[^ ^55^, ^56^ ^]^ In addition, unlike commercial SESAMs, which operates in a reflective fashion, the 2D material‐based SAs can be integrated into a fiber or solid‐state laser for a more integrated and stable pulsed laser. In this section, we briefly review recent results of short‐pulse laser generations and light emitters enabled by a few representative 2D materials, covering graphene, TMDs, BP, MXenes, and vdWs heterostructures in turn. Pulsed laser based on 2D materials was first realized by Bao et al. in 2009. They demonstrated the use of atomic‐layer graphene deposited onto the end face of a fiber connector as SAs in a fiber laser for the generation of ultrashort mode‐locked soliton pulses at 1567 nm (**Figure** **5**a),^[^ ^7^ ^]^ with a pulse duration of 756 fs, a repetition rate of 1.79 MHz and a maximum average output power up to 2 mW (Figure 5b,c). Following that, using the same saturated absorption structure, Sun et al. demonstrated a passively mode‐lock erbium‐doped fiber laser working at 1559 nm,^[^ ^57^ ^]^ with a 5.24 nm spectral bandwidth and 460 fs pulse duration. Since then, graphene‐based saturable absorbers (GSA) has been used in lasers of various structures to achieve pulse output with different parameters including wavelength, repetition rate, and pulse duration, as well as different principles including passively mode‐locked,^[^ ^58^ ^]^ actively mode‐locked,^[^ ^59^ ^]^ passively Q‐switched,^[^ ^60^ ^]^ and actively Q‐switched.^[^ ^61^ ^]^ More detail, Bogusławski et al.^[^ ^59^ ^]^ reported an active mode‐locked laser that achieves electronic control repetition rate of generated pulses by using a graphene‐based electro‐optic modulator (GEOM). The active mode‐locking and active harmonic mode‐locking of the erbium‐doped fiber laser with output pulse duration of 1.44 ps and pulse energy of 844 pJ are achieved by the combination of the active mode‐locking technique and the intracavity nonlinear pulse compression effect. Yao et al.^[^ ^60^ ^]^ demonstrated single‐frequency passively Q‐switched in a compact graphene‐coated DFB fiber laser with a pulse energy of up to 10 nJ and a linewidth of hundreds of kHz. The pulses are near transform‐limited and with kHz repetition rates. Li et al.^[^ ^61^ ^]^ reported the first actively Q‐switched lasers with electrically tunable output parameters, such as repetition rate, pulse duration, and pulse energy by using a GEOM.

With the successful demonstration of ultrafast lasers based on graphene as a saturation absorbers (SAs), more and more ultrafast lasers based on TMDs, BP, and MXenes including Q‐switching and mode‐locking lasers have been successfully demonstrated in the NIR and MIR region.^[^ ^62^, ^63^, ^64^ ^]^ The first pulsed laser based on TMDs material was reported by Wang et al.^[^ ^65^ ^]^ using the MoS_2_‐based SAs. This solid‐state Q‐switched pulse laser work at wavelengths of 1.06, 1.42, and 2.1 µm based on laser crystals of Nd:GdVO_4_, Nd:YGG, and Tm:Ho:YGG, respectively. Soon after, Xia et al.^[^ ^66^ ^]^ introduced the few‐layer MoS_2_ into the fiber laser to achieve a stable passive Q‐switched fiber laser with a pulse duration of 1.92 µs at 1560.5 nm. The output pulse repetition rate of the laser can be widely adjusted from 28.6 to 114.8 kHz by changing the input pump power from 42 to 204 mW. Using the same SAs and structure, Zhang et al.^[^ ^67^ ^]^ demonstrated an ultrafast mode‐locked fiber laser with stable picosecond pulses, that can be tuned from 1535 to 1565 nm. Other TMDs, such as WS_2_, MoSe_2_, WSe_2_, and WTe_2_, exhibit similar nonlinear optical (NLO) absorption, and SAs based on these materials are also used in Q‐switched and mode‐locked pulsed lasers. Because of the broadband ultrafast saturable absorption of TMDs, Luo et al.^[^ ^55^ ^]^ and Liu et al.^[^ ^68^ ^]^ demonstrated passively Q‐switched all‐fiber laser in visible (635 nm) and communication bands, respectively, by deposited few layers TMDs (including MoS_2_, MoSe_2_,WS_2_, and WSe_2_) on the end face of the fiber to form a SAs (Figure 5d–f). The modulation depths of WSe_2_ and MoSe_2_ SAs and the SNRs of the output pulse laser can be effectively improved by optimizing the film uniformity and regulate the film thickness. In addition to depositing TMDs on the end face of fiber, some other saturated absorption structures, including D‐shaped fibers and tapered fibers, have also been studied in recent years. Typically, Koo et al.^[^ ^69^ ^]^ deposited WTe_2_ on the D‐shaped fiber to form a SAs, demonstrated that a mode‐locked femtosecond pulses with a modulation depth of 2.85%, a repetition rate of 13.98 MHz and a duration of 770 fs at 1556.2 nm. Liu et al.^[^ ^70^ ^]^ deposited WS_2_ on the surface of the tapered fiber to form a SAs, presenting an all‐fiber mode‐locked erbium‐doped fiber (EDF) laser with a modulation depth of 25.48% and a duration of 246 fs.

Due to similar bandgap structures, single‐layer or few‐layer BP displays similar nonlinear optical properties as that of 2D TMDs. Nevertheless, the bandgap of BP in few‐layer stacks is smaller than TMDs, that mean a much larger bandwidth for saturable absorption. Therefore, BP has shown to be an excellent MIR SAs for either Q‐switching or mode‐locking pulse generation in spectral region of 2 µm and longer.^[^ ^71^ ^]^ Mu et al.^[^ ^72^ ^]^ applied an optically transparent polymer matrix on BP to form a composite SAs, and demonstrated a highly stable Q‐switched fiber laser pulse generation with a modulation depth of 10.6% and a single pulse energy of 194 nJ, as shown in Figure 5g–i. Through the mode‐locking of solid‐state lasers based on Pr:LiGdF4, Nd:GdVO4, and Tm:Ho:YGG crystals, respectively, Zhang et al.^[^ ^73^ ^]^ verified the broadband saturated absorption of BP from 639 nm, 1060 nm to 2100 nm.

Recently, the broadband saturable absorption properties from 800 to 1800 nm were systematically characterized by Jiang et al.^[^ ^40^ ^]^ They found the modulation depth of the MXene samples increase with the incident laser intensity. The highest modulation depth of MXene based saturable can be as high as 40%. Moreover, multiphoton absorption might be induced under intense light illuminations, which leads to the decrease of optical transmission. By using MXene Ti_3_C_2_ as the saturable absorber, mode‐locked laser operations have been realized at 1.06 and 1.55 µm with femtosecond pulse durations in fiber resonators. Recently, MXene Ti_3_C_2_ ink has been prepared for versatile inkjet‐printing on varies substrates, including side‐polished fiber (D‐shape fiber) and gold mirror, that can be compatible to both fiber and solid‐state resonators. By means of printed MXene saturable absorbers, ultrabroad band pulsed lasers from 1.06 to 2.8 µm have been successfully achieved with pulse duration down to 100 fs.^[^ ^74^ ^]^ Besides, the use of MXene based saturable absorbers has expanded to solid‐state and ceramic lasers.^[^ ^75^, ^76^, ^77^ ^]^ Apart from ultrafast laser generations,^[^ ^40^, ^74^, ^76^, ^78^, ^79^ ^]^ MXenes can be combined with C_60_ to realize a nonreciprocal transmission photonic diode, due to the reverse saturable absorption properties of C_60_.^[^ ^80^ ^]^

### 4.2 Light Emitters

**Figure 6:**
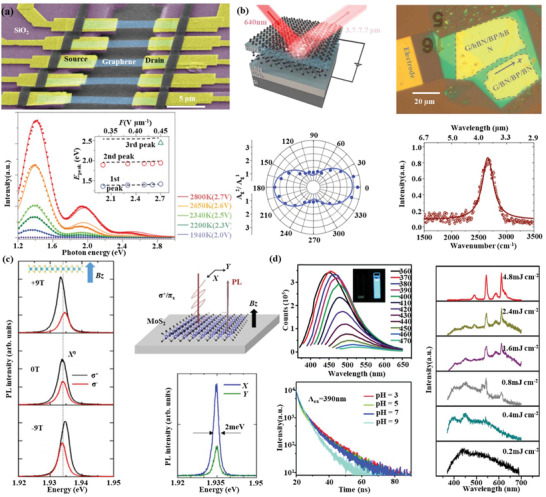
2D material‐based light emitters. a) Suspended graphene for enhanced photon emission at visible regime. Reproduced with permission.^[^ ^83^ ^]^ Copyright 2015, Nature Publishing Group. b) Mid‐infrared black phosphorus emitter under the excitation of 640 nm. Reproduced with permission.^[^ ^90^ ^]^ Copyright 2020, American Association for the Advancement of Science. c) Valley transition in a MoS_2_ monolayer in the presence of the magnetic field. Reproduced with permission.^[^ ^87^ ^]^ Copyright 2017, American Physical Society. d) Photoluminescence wavelengths shifted by the excitation wavelengths (left‐up), Reproduced with permission.^[^ ^95^ ^]^ Copyright 2018, Royal Society of Chemistry, the PL lifetime of MXene QDs at different pH values (left‐down) Reproduced with permission.^[^ ^96^ ^]^ Copyright 2018, Royal Society of Chemistry, and the emerging of the random laser as the increase of pump power (right), Reproduced with permission.^[^ ^92^ ^]^ Copyright 2019, Wiley‐VCH.

2D materials feature different energy structures and bandgap; the direct transitions of carriers from the excited states to ground states in the presence of photons offer the stage of wavelength transition and efficient phosphorous and their corresponding applications. Graphene, as the prior of 2D materials, known for featuring a Dirac‐cone like zero bandgap, indicates it is unpromising as light emitter. Yet, the applied bias electric field on graphene incurred the hot‐electron localization with temperature over 1500 K, and thereafter thermal radiation as a form of infrared emission.^[^ ^81^, ^82^ ^]^ Suspending the graphene can significantly increase the spatial localization of hot electrons, and thus the thermal radiation efficiency. Bright visible light emission has been achieved in micrometer‐sized graphene‐silicon chips with hot electrons around 2800 K (**Figure** **6**a).^[^ ^83^ ^]^ Another pathway to achieve photon emission is bandgap engineering, namely, by opening the zero bandgap to a direct one. One simple strategy is by reducing the graphene size to quantum dot (QD) regime, where the nontrail Dirac‐cone disappeared. Single‐photon emission from graphene quantum dots has been demonstrated at room temperature.^[^ ^84^ ^]^ Furthermore, the energy bandgap of graphene QDs can be tuned by chemical decorations or functionalization of the QDs' edges.^[^ ^85^, ^86^ ^]^ Likewise, other 2D materials have followed the abovementioned principles and have been used to demonstrate light emission from visible to the mid‐infrared regime. 2D materials, like TMDs, BP, boron nitride, featuring direct bandgaps can ignore the bandgap opening, yet require certain atomic layers or strains to tune the indirect bandgap to be directly, or increase the emission efficiency and adjust the wavelength. For example, the quantum yield of monolayer MoS_2_ can be two‐order superior to the bilayer one, due to the direct to indirect bandgap transition.^[^ ^2^ ^]^ In TMDs, the Coulomb attraction between excited electrons and holes results in the formation of the exciton, trion, and biexciton, which are normally sensitive to the environmental temperature. Besides, the inversion symmetry breaking in monolayer TMDs offers the possibility of valley emission at K (and −K) point with circular dichroism (Figure 6c).^[^ ^87^ ^]^ Black phosphorus is outstanding among the 2D materials due to its large layer‐dependent energy bandgap (0.3–2 eV) from visible to mid‐infrared. Indeed, the emission spectra of BP have been achieved to cover most of this expectation.^[^ ^88^, ^89^ ^]^ Recently, widely tunable mid‐infrared light emission (3.7–7.7 µm) has been realized from one ≈10 nm thick BP flake. Besides, the emission shows linear polarization along the armchair direction under the biasing field (Figure 6b).^[^ ^90^ ^]^ Recently, MXene as a novel 2D material member with alluring optoelectronics properties has attracted quite lots of attention.^[^ ^91^ ^]^ Thanks to the chemical richness of MXene composition, the band structure can be largely tuned. Besides, the strategies like particle size reduction,^[^ ^92^ ^]^ chemical decoration,^[^ ^93^, ^94^ ^]^ doping,^[^ ^95^ ^]^ pH value,^[^ ^96^ ^]^ electric field,^[^ ^97^ ^]^ and strains^[^ ^98^ ^]^ have either been experimentally demonstrated or been theoretically predicted. The highest photoluminescence quantum yield of 18.7% was obtained in nitrogen‐doped Ti_3_C_2_ quantum dots.^[^ ^95^ ^]^ The obtained emission frequency has well covered the visible regime, and white random laser using V_2_C MXene QDs has recently been realized, as shown in Figure 6d.^[^ ^92^ ^]^

### 4.3 Nonlinear Optical Devices and Frequency Converters

Nonlinear optics and frequency conversion are also an important topic in information science. Traditional nonlinear materials have demonstrated excellent applications of nanophotonics and nonlinear plasmas in the field of nonlinear nanophotonics. But restricted by the relatively low nonlinear polarizability of the traditional nonlinear materials and the existing manufacturing and integration process, traditional nonlinear material can no longer satisfy the current demand for quantum integration and photonic integrated circuits (PICs). Therefore, it is of great significance for the development of photonics and optoelectronics to find new materials that are easy to integrate on chip and have higher nonlinear response.

For the past few years, the NLO properties (e.g., large optical nonlinearity,^[^ ^11^ ^]^ strong exciton effect^[^ ^99^ ^]^) of 2D materials have gathered huge interest,^[^ ^14^ ^]^ and have successfully demonstrated second‐harmonic generation (SHG) in TMDs,^[^ ^100^, ^101^ ^]^ third‐harmonic generation (THG), and four‐wave mixing (FWM) in graphene,^[^ ^102^ ^]^ TMDs,^[^ ^103^, ^104^ ^]^ BP,^[^ ^27^, ^105^ ^]^ and MXenes,^[^ ^106^ ^]^ and high harmonic generation (HHG) in graphene,^[^ ^107^ ^]^ TMDs,^[^ ^108^ ^]^ and BP,^[^ ^109^ ^]^ which shows the fundamental differences between novel 2D material‐based nonlinear devices and traditional bulk material‐based nonlinear devices.^[^ ^11^, ^99^ ^]^ There are several nonlinear properties about 2D materials worth mentioning. First, there is almost no second‐order but a strong third‐order nonlinear optical effects in graphene due to the centrosymmetric crystal structure of graphene. Second, second‐order (e.g., SHG) and other even‐order nonlinear optical effects are allowed in TMDs with odd numbers of layers which are not present in TMDs with even number of layers due to the inversion symmetry in the crystal structure, unless breaking its centrosymmetric.^[^ ^110^ ^]^ Third, the centrosymmetric crystal structure of BP only allows third‐order nonlinearity, but its strong anisotropy and energy band structure of dependent layer will lead to very interesting nonlinear optical effects. Here, we give a brief review of the recent works.

**Figure 7:**
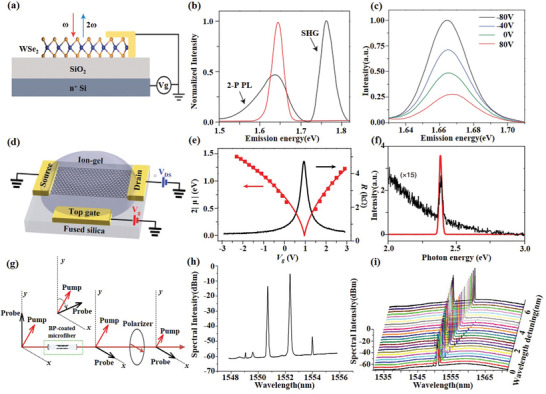
2D material‐based nonlinear devices and frequency convertors. a) Schematic of gated monolayer WSe_2_. Excitation at ω (red arrow) generates second‐harmonic radiation at 2ω (blue arrow). b) Emission spectrum for excitation at 0.83 eV (red curve) dominated by SHG, and 0.88 eV (black curve), showing SHG at 1.76 eV and two‐photon‐induced photoluminescence (2‐P PL) from the exciton. c) SHG spectra on resonance with the exciton at selected gate voltages. Reproduced with permission.^[^ ^101^ ^]^ Copyright 2015, Nature Publishing Group. d) Schematic of an ion‐gel‐gated graphene monolayer on a fused silica substrate covered by ion‐gel and voltage biased by the top gate. e) Measured graphene resistance (*R*) as a function of gate voltage *V*_g_ (black curve), the peak of which refers to the zero chemical potential (*µ* =  0). f) Measured THG spectra by a normally incident femtosecond input pulse at 1566 nm from graphene gated at *µ* =  0 (black curve, magnified by 15 times) and *µ* = −0.74 eV (red curve). Reproduced with permission.^[^ ^102^ ^]^ Copyright 2018, Springer Nature. g) Schematic diagram of the optical Kerr switcher based on BP‐coated microfiber. h) Typical output FWM spectrum obtained after the BP‐coated microfiber with two newly converted idlers at 1549.05 and 1554.0 nm. i) Output FWM spectra against wavelength detuning. Reproduced with permission.^[^ ^27^ ^]^ Copyright 2017, Wiley‐VCH.

As mentioned above, in the absence of symmetry breaking, graphene has almost no second‐order nonlinear effects. Therefore, the research on graphene nonlinearity focuses more on THG and FWM based on third‐order nonlinearity and HHG. Jiang et al.^[^ ^102^ ^]^ demonstrated electrically tunable THG and FWM in graphene by tuning of chemical potential in graphene by ion‐gel gating (**Figure** **7**d). The result shows that THG and the sum‐frequency FWM have a strong enhancement in heavily doped graphene, but the difference‐frequency FWM is the opposite, as shown in Figure 7e,f. Yoshikawa et al.^[^ ^107^ ^]^ found that HHG signals in graphene were significantly enhanced by elliptically polarized excitation compared with the linear polarization excitation by studying the 5th, 7th, and 9th harmonics in graphene. These works show that the ability to tune the nonlinear responses of graphene can potentially enable various electrically tunable nonlinear optical devices for future, such as the gate‐tunable frequency comb.^[^ ^9^ ^]^

Unlike graphene, we can obtain second‐order nonlinearity by controlling the number of layers of TMDs. Since the magnitude of the second‐order nonlinearity of TMDs depends on exciton resonance, Seyler et al.^[^ ^101^ ^]^ demonstrated the modulability of the SHG response in monolayer WSe_2_ using electrical doping, increasing the SHG intensity by approximately four times with 160 V gate voltage, as shown in Figure 7a–c . Afterward, the ultrastrong SHG and THG optical nonlinearities in monolayer MoS_2_ was reported by Säynätjoki et al.^[^ ^103^ ^]^ They found that the third harmonic is 30 times stronger than the second and fourth harmonic, and the third harmonic generation efficiency of the single‐layer MoS_2_ is about three times that of graphene because of the larger χ^(3)^. Based on the third‐order nonlinearity, the FWM‐induced NLO effects in TMDs were first investigated in MoS_2_ thin films and MoS_2_‐graphene heterostructures with NLO microscopy.^[^ ^104^ ^]^ Furthermore, Langer et al.^[^ ^108^ ^]^ demonstrated HHG with odd orders up to 47 with a THz‐driven scheme in the same WSe_2_, because the mechanism of HHG in MoS_2_ (including other TMDs) is different from that in zero‐gap graphene due to the finite bandgap.

Similar and different from graphene, the third‐order nonlinearity of BP is stronger than that of graphene and has anisotropy. Rodrigues et al.^[^ ^105^ ^]^ observed and studied third harmonic generation in black phosphorus, the results show that both the direct mechanical exfoliation and the laser thinning method can obtain a third‐order susceptibility drastically and resonantly increases that is one order of magnitude higher than that of graphene. Furthermore, the third‐order nonlinear‐induced four‐wave mixing has been experimentally verified on BP by coating few layer BP on the surface of the microfiber (Figure 7g–i).^[^ ^27^ ^]^ In addition, BP has the ability to produce HHG which is anisotropic.^[^ ^109^ ^]^ These results show that BP has great potential in broadband all‐optical nonlinear processors.

Compared with other 2D materials, there are few researches on the nonlinear optical properties of MXenes, mainly focusing on the third‐order nonlinear optical effects. Due to the good third‐order nonlinear optical properties, Song et al.^[^ ^106^ ^]^ reported an FWM‐based all fiber wavelength convertor by depositing MXene Ti_3_C_2_T*_x_* on a microfiber. It shows an excellent nonlinear optical response at the telecommunication band with the FWM conversion efficiency of −59 dB and the modulation speed of 10 GHz.

### 4.4 Modulators and Switchers

According to different modulation principles (e.g., electro‐optic effect, acousto‐optic effect, magneto‐optic effect, Franz–Keldysh effect, quantum well Stark effect, carrier dispersion effect), optical modulators can be divided into electro‐optic, acousto‐optic, magneto‐optica, all‐optical modulators, etc. Traditional optical modulators are mainly based on bulk materials (e.g., lithium niobate (LiNbO_3_), gallium arsenide (GaAs)), which are difficult to integrate, and the modulation bandwidth is limited to dozens of gigahertz. In contrast, 2D materials have shown their unique advantages in optical modulation, opening new opportunities for smaller and faster optical modulators and related devices. First, the atomic thickness makes it easy to combine with existing optical structures, as mentioned in Section 3, for smaller volumes and greater integration.^[^ ^111^ ^]^ Second, the ultra‐broadband optical response makes it have the application potential of wideband optical modulation from ultraviolet to terahertz.^[^ ^6^ ^]^ Third, the ultrafast carrier relaxation time and the strong light–material interaction of 2D materials make them possible to achieve modulation rates above 200 GHz^[^ ^112^ ^]^ and modulation depths approaching 100%.^[^ ^113^ ^]^ In this section, we briefly summarize recent results of optical modulators and switchers enabled by a few representative 2D materials, covering graphene, BP, and MXenes.

Graphene‐based optical modulators have recently attracted much attention because of their characteristic ultrafast and broadband response. Various graphene‐based modulators including electro‐optic,^[^ ^114^, ^115^ ^]^ all‐optical,^[^ ^112^ ^]^ thermo‐optic,^[^ ^116^ ^]^ and other less‐explored modulators^[^ ^117^ ^]^ have been demonstrated covering the visible,^[^ ^118^ ^]^ infrared,^[^ ^114^ ^]^ and terahertz^[^ ^119^ ^]^ range in recent years.

**Figure 8:**
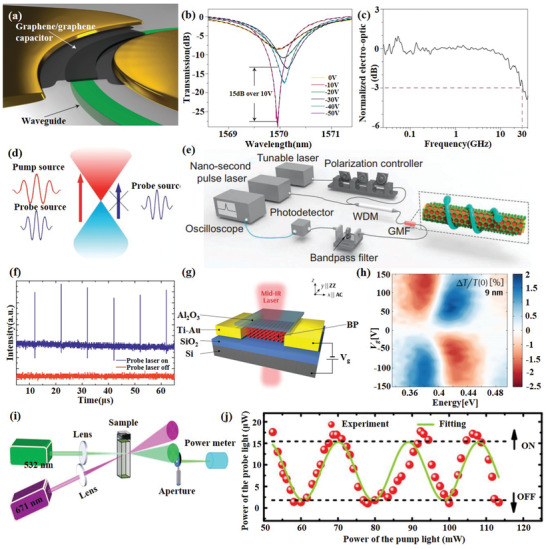
2D material‐based modulators and switchers. a) Schematic of the modulator consisting of a graphene/graphene capacitor integrated along a ring resonator. b) Transmission spectra at various applied d.c. voltages. c) Electro‐optic frequency response with 30 GHz bandwidth. Reproduced with permission.^[^ ^123^ ^]^ Copyright 2015, Nature Publishing Group. d) Schematic illustration of the all‐optical modulation mechanism. e) Schematic illustration of the all‐optical modulation measurement system. f) Time‐domain response of the GMF modulator. Reproduced with permission.^[^ ^124^ ^]^ Copyright 2015, Nature Publishing Group. g) Schematic illustration of the BP modulator. h) The modulation level measured as functions of energy and gate bias for 9 nm thick BP. Reproduced with permission.^[^ ^111^ ^]^ Copyright 2017, American Chemical Society. i) Ti_3_C_2_T*_x_* MXene Ns‐based all‐optical switcher. j) The probe light modulated by the pump light to realize “ON” and “OFF” modes in all‐optical switcher. Reproduced with permission.^[^ ^41^ ^]^ Copyright 2018, Wiley‐VCH.

Electro‐optic modulators (EOM) are the most commonly used modulator in practical applications. The first graphene‐based modulators reported by Liu et al.^[^ ^114^ ^]^ in 2011 successfully demonstrated waveguide‐integrated graphene‐based electro‐absorption modulator with modulation frequency over 1 GHz and a broad operation spectrum ranging from 1.35 to 1.6 mm by electrically tuning the Fermi level of the graphene sheet. Afterward, Hu et al.^[^ ^120^ ^]^ increased the modulation speed to 10 Gb s^−1^ with the insertion loss of 3.8 dB at 1580 nm and a low drive voltage of 2.5 V. However, phase modulators are as important as amplitude modulators. More recently, a graphene‐based phase modulator^[^ ^121^ ^]^ was demonstrated by integrating graphene bipolar junction transistor into a Mach–Zehnder interferometer(MZI) configuration. It presents a modulation speed of 10 Gb s^−1^, a phase‐shifter length of 300 µm, extinction ratio of 35 dB, and a modulation efficiency of 0.28 V cm^−1^ at 1550 nm. Furthermore, Gao et al.^[^ ^122^ ^]^ demonstrated a GEOM based on a graphene‐hBN heterostructure integrated with a planar photonic crystal (PPC) cavity. The PPC cavity greatly enhances the interaction of light–matter with a modulation depth of 3.2 dB making efficient electrical tuning of cavity reflection possible. The electro‐optic graphene modulators reported above have been limited in bandwidth to a few gigahertz. In order to achieve a greater bandwidth, Phare et al.^[^ ^123^ ^]^ demonstrated a GEOM based on resonator loss modulation in a critically coupled by pasting a piece of 30 µm length graphene film on the surface of a waveguide(**Figure** **8**a), operating with a 30 GHz bandwidth and a modulation efficiency of 15 dB per 10 V, as shown in Figure 8b,c.

All‐optical modulators can provide an ultrafast response based on optical nonlinear effects by using a switching beam (e.g., ultrafast pulse) to control the propagation of another beam (e.g., signal light). In 2014, Li et al.^[^ ^112^ ^]^ presented on graphene‐clad microfiber (GCM) all‐optical modulator, achieved a modulation depth of 38% and response time of about 2.2 ps which is only restricted by the intrinsic carrier relaxation time of graphene, through constructing pump‐detection delay system for all‐optical modulation, the biggest modulation rate for the Gaussian pulse up to 200 GHz. One way to increase the modulation depth is to increase the interaction distance between light and graphene. Based on this, a stereo graphene–microfiber all‐optical modulator^[^ ^124^ ^]^ was demonstrated with a modulation depth up to 7.5 dB by spirally winding the microfiber on a glass rod deposited with graphene on the surface to achieve an ultralong graphene–microfiber (GMF) interaction, as shown in Figure 8d–f. More recently, by integrating graphene‐coated microfibers into one arm of MZI structure, an all‐optical graphene phase modulator based on the optical Kerr effect was reported Yu et al.^[^ ^125^ ^]^ with a 4.6‐fold improvement of modulation depth compared with amplitude modulation.

Owing to its high intrinsic thermal conductivity, graphene is very attractive for thermo‐optic modulators and graphene‐based thermo‐optic modulators have also been reported in recent years. Using the same structure as ref. [100], Gan et al.^[^ ^116^ ^]^ proposed an all‐fiber phase modulators based on graphene's photothermal effect. Heating the graphene by 980 and 1540 nm pump light can effectively change the refractive index of the fiber and then change the interference phase shift of the MZI. The phase shift over 21 π is possible linear slopes of 0.091 and 0.192 π mW^−1^, which can be used as an all‐optical switcher. In addition, graphene is also used in magneto‐optic^[^ ^117^ ^]^ and acousto‐optic^[^ ^126^ ^]^ modulators.

Optical modulation techniques in the visible and near‐infrared regions are well established, but modulation of terahertz (THz) light is still a challenge we need to face. Since the conductivity of graphene can be tuned by the gate voltage, it is widely used in terahertz modulators. Utilizing the evanescent wave interacting with graphene during total internal reflection. Liu et al.^[^ ^119^ ^]^ designed a broadband THz modulator with a modulation depth greater than 90% between 0.15 and 0.4 THz by applying a gate voltage between 0.1 and 2 V to a graphene layer to change the attenuation during total internal reflection. Last year, Chen et al.^[^ ^113^ ^]^ of the same research group further improved the modulation depth to over 99.3% and extending modulation wavelength range to 0.5–1.6 THz by varying the conductivity of graphene to control the Brewster angle.

So far, modulators based on TMDs are barely reported. But multilayer BP has a broad application prospect for mid‐IR optical modulation. Strong field‐effect tuning of its bandgap has been observed in BP, and its infrared optical response has been measured. In 2016, Lin et al.^[^ ^127^ ^]^ demonstrated a BP‐based electroabsorption modulator and verified the feasibility of BP film as an electro‐optic material for modulating the MIR frequency. Controlled by the quantum‐confined Franz–Keldysh (QCFK) effect and carrier‐induced Burstein–Moss shift (BMS), the absorption spectra of BP may undergo red, blue, or bidirectional shift depending on doping level, wavelength, and BP film thickness. Meanwhile, Whitney et al.^[^ ^128^ ^]^ reported the infrared optical response of thin black phosphorus under field‐effect modulation. Due to the combination of ambipolar BMS and QCFK behavior, the transmitted extinction modulation amplitude is greater than 2%. These two works indicate the potential application of black phosphorus in infrared photoelectricity modulators. Soon afterward, a BP‐based MIR electro‐optic modulator with higher modulation depth (5 dB) and smaller size (100 µm) was demonstrated by Peng et al.^[^ ^111^ ^]^ (Figure 8g,h). To date, all reported BP‐based modulators utilize the feature of black phosphorus field effect optoelectronic modulation, and there is no report on other modulation methods based on black phosphorus, so BP‐based modulator is still under the development.

Recently, solution‐based spatial self‐phase modulation (SSPM) techniques have been used to characterize the nonlinear refractive index of a bunch of 2D materials.^[^ ^129^, ^130^ ^]^ Wu et al.^[^ ^41^ ^]^ found the nonlinear refractive index of Ti_3_C_2_T*_x_* MXene can be as large as 10^−4^ cm^2^ W^−1^ in the visible optical regime, which is higher than the previously determined values via Z‐scan method.^[^ ^40^ ^]^ This discrepancy may originate from the different characterization method and sample preparations. Nevertheless, by utilizing the excellent optical Kerr effect, a Ti_3_C_2_T*_x_* MXene nanosheets dispersion based all‐optical switcher realizing alternative “ON” and “OFF” modulations have been well demonstrated, as shown in Figure 8i,j.

### 4.5 Photodetectors and Imaging Devices

High‐speed, broadband and high‐sensitive photodetectors that can convert optical signals into electrical ones are of great practical significance for communication, sensing, and digital imaging. Currently, high‐performance photodetectors are mainly made of crystal silicon (Si) with a detection range from visible light to near infrared (NIR). In addition, using InGaAs and related heterostructures can achieve longer wavelength photodetectors beyond the detection limit of Si‐based products. However, these detectors often have serious shortcomings, such as large amount of materials, expensive manufacturing process, strict control of manufacturing conditions, and strict operational requirements. The appearance of photodetectors based on 2D materials makes up for the deficiency of current Si‐based technology, with the advantages of high transparency, strong light substance interaction, good flexibility, and easy processing, which provide a broad prospect for the realization of high‐performance photodetectors.

The first photodetector based on 2D material was reported by Xia et al. in 2009.^[^ ^131^ ^]^ Compared to traditional semiconductor photodetectors, graphene‐based photodetectors demonstrated great advantages in terms of bandwidth, possibly exceed 500 GHz. However, although pure graphene‐based photodetectors show high performance in high frequency devices, their photoresponsivity has so far been limited to tens of mA W^−1^ due to the fast carrier dynamics and low light absorption by single layer graphene (SLG). Therefore, several methods are developed to improve the photoresponsivity.

**Figure 9:**
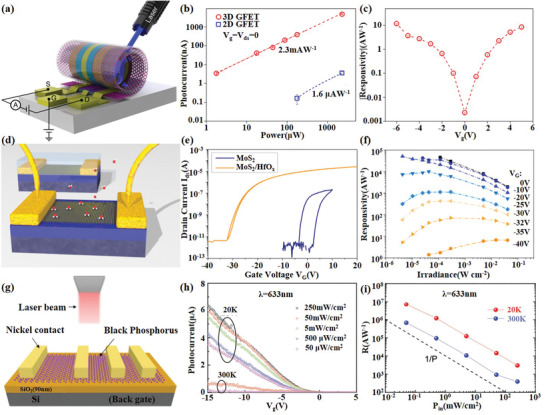
2D material‐based photodetectors and imagers. a) Schematic of the 3D GFET photodetector. b) The photocurrent at different incident ultraviolet laser power. c) The photoresponsivity at different *V*_g_. Reproduced with permission.^[^ ^136^ ^]^ Copyright 2019, American Chemical Society. d) A 3D sketch of TMDs FETs. e) Transfer curve IDS−VG before and after ALD encapsulation. f) Power‐dependent responsivity for a wide dynamic range at *V*_G_ = −40 V until 0 V. Reproduced with permission.^[^ ^137^ ^]^ Copyright 2015, American Chemical Society. g) Cross‐section view of the BP‐based photodetector. h) Photogenerated current in backgate‐voltage‐dependent photodetection under different laser power density. i) The photoresponsivity of the device at different incident laser power in 633 nm. Reproduced with permission.^[^ ^141^ ^]^ Copyright 2016, Wiley‐Blackwell.

One way is to integrate graphene with microcavities,^[^ ^132^ ^]^ waveguide,^[^ ^133^ ^]^ and plasmonic,^[^ ^134^ ^]^ but these methods restrict photodetection to narrowband. For example, Echtermeyer et al.^[^ ^134^ ^]^ utilized surface plasmon polariton generated by a plasmonic grating to transfer the collected photons to the junction of the metal graphene metal photodetector, realizing a 400% enhancement of responsivity. The other way is to improve responsivity significantly through integrating QDs in the light absorption layer. For example, a highly sensitive QD/graphene hybrid photodetector is demonstrated by Hu et al. in this year.^[^ ^135^ ^]^ When light is incident on the quantum dots, the photogenerated carriers in the quantum dots will increase the carrier concentration of graphene, which will bring the gain of the current, resulting an ultrahigh responsivity over 10^9^ A W^−1^ and fW light detectivity. However, the improved responsivity comes at the expense of spectral bandwidth because light absorption occurs in the quantum dots. In order to simultaneously realize photodetectors with ultra‐wideband and high responsivity, in this year, Deng et al.^[^ ^136^ ^]^ rolled up 2D GFETs to form 3D tubular GFETs (**Figure** **9**a) to balance photoresponsivity, spectral range, and bandwidth. This novel 3D GFET photodetector structure significantly enhanced optical absorption in two aspects, one is to form a natural cavity to enhance the light field, and the other is to increase the interaction area between light and graphene (Figure 9b). While maintaining the intrinsic ultrafast and ultra‐broadband photoelectronic properties of graphene, it significantly improved the photoresponsivity from ultraviolet to terahertz (THz) regions.

Beyond graphene, single‐ or few‐layer TMD also shows great potential in photodetection because of the direct bandgap, large carrier mobility, high‐current ON/OFF ratio, and strong photoluminescence. So far, TMD‐based photodetectors have operated at wavelengths ranging from ultraviolet to near infrared because of their wide bandgaps.

In 2015, Kufer et al.^[^ ^137^ ^]^ made efforts to get a much higher responsivity and a much shorter response time, they found that encapsulation with atomic‐layer‐deposited HfO_2_ can significantly enhance both the electronic and optoelectronic performance of monolayer or few‐layer MoS_2_ phototransistors, as shown in Figure 9d. Responsivity and response time can be tuned by several orders of magnitude with responsivity from 10^4^ to 10 A W^−1^ and response time from 10 s to 10 ms via controlling the gate voltage (Figure 9f). It reveals the enormous potential of encapsulated MoS_2_ devices as ultrathin visible light detectors. Meanwhile, Wang et al.^[^ ^138^ ^]^ demonstrated a monolayer MoS_2_ photodetector using two‐pulse photovoltage correlation (TPPC) technology with an inherent response time as short as 3 ps, meaning that the optical detection bandwidth can reach 30 GHz. TPPC technique contributes to promote the photoresponse because it is only sensitive to the total population of photoexcited carrier, including both bound and free carriers. Besides MoS_2_, photodetectors based on other TMDs including MoSe_2_, WS_2_, WSe_2_, MoTe_2_ materials have also been reported in recent years. Typically, Jo et al.^[^ ^139^ ^]^ demonstrated a high‐performance WSe_2_‐based optoelectronic transistor with a responsivity as high as 1.27 × 10^6^ A W^−1^ and fast response times of 2.8 ms by inserting an h‐BN layer under WSe_2_ channel to further improve the performance and provide a charge‐free environment which can greatly improve carrier mobility.

The narrow bandgap renders few‐layer BP an appealing candidate for NIR photodetection, complementing the detection gap between zero‐bandgap graphene and large‐bandgap TMDs. In 2015, Youngblood et al.^[^ ^140^ ^]^ demonstrated a multilayer BP‐based near‐infrared telecom band photodetector by integrating on a silicon photonic waveguide, which has a very low dark current and a high response bandwidth of over 3 GHz. The responsivity can be tuned from 135 to 657 mA W^−1^ by varying the black phosphorus thickness from 11.5 to 100 nm. Subsequently, the relationship between the optical response of BP‐based photodetectors and temperature, incident laser power density, photon energy, and channel length was systematically studied by Huang et al.^[^ ^141^ ^]^ They extended the detection wavelength to 400–900 nm (Figure 9g) and greatly improved the photoresponsivity to 7 × 10^6^ A W^−1^ at 20 K and 4.3 × 10^6^ A W^−1^ at 300 K in a broadband spectrum (Figure 9i). More recently, the detected wavelength range is refreshed to the mid‐infrared region from 3.68 to 4 µm.^[^ ^142^ ^]^ Integration of silicon‐on‐insulator (SOI) waveguides with BP photodetectors achieved a responsivity of 23 A W^−1^ at 3.68 µm and 2 A W^−1^ at 4 µm at bias of 1 V.

van der Waals materials also show high performance in photodetectors, and more and more photodetectors based on van der Waals materials have been reported in recent years including graphene‐TMDs,^[^ ^143^, ^144^ ^]^ graphene‐BP,^[^ ^145^ ^]^ TMDs–TMDs,^[^ ^146^ ^]^ TMD‐BP,^[^ ^147^ ^]^ and BP‐hBN.^[^ ^148^ ^]^ Brief introduction, Xu et al.^[^ ^144^ ^]^ demonstrated a transparent photodetector based on novel few layer graphene/MoS_2_ heterostructure with responsivity of 12.3 mA W^−1^ and detectivity of 1.8 × 10^10^ Jones at 532 nm. Liu et al.^[^ ^145^ ^]^ demonstrated a graphene‐BP heterostructure based highly efficient and air‐stable infrared (1550 nm) photodetector with an ultrahigh photoresponsivity of 3.3 × 10^3^ A W^−1^, a photoconductive gain of 1.13 × 10^9^ and a rise time of about 4 ms. Xue et al.^[^ ^146^ ^]^ demonstrated a telecommunication wavelength photodetector based on MoSe_2_/WSe_2_ heterostructure with a photoresponsivity up to 127 mA W^−1^. Deng et al.^[^ ^147^ ^]^ demonstrated a gate‐tunable MoS_2_‐BP heterostructure‐based p–n diode, in which BP is p‐doped and monolayer MoS_2_ is n‐doped. It showed a photodetection responsivity of 418 mA W^−1^ at 633 nm, which is nearly 100 times higher than the pure BP phototransistor. Viti et al.^[^ ^148^ ^]^ embedded a BP flake in multilayered hBN crystals to devise hBN/BP/hBN heterostructure THz photodetectors operating at 0.3–0.65 THz with high optical response, and an extremely good time‐dependent electrical stability.

### 4.6 Plasmonic Generators

On the surface of the ultrathin gold‐like and semiconductor layer, the photon‐induced high‐efficiency electron cluster resonance is called surface plasmon polaritons (SPP). Different from the traditional metal SPP, 2D material‐based SPP has excellent electrical tunability, high field confinement, long lifetimes, and strong light–matter interactions. In this section, we focus on the state‐of‐art of plasmonic generators based on 2D Materials.

**Figure 10:**
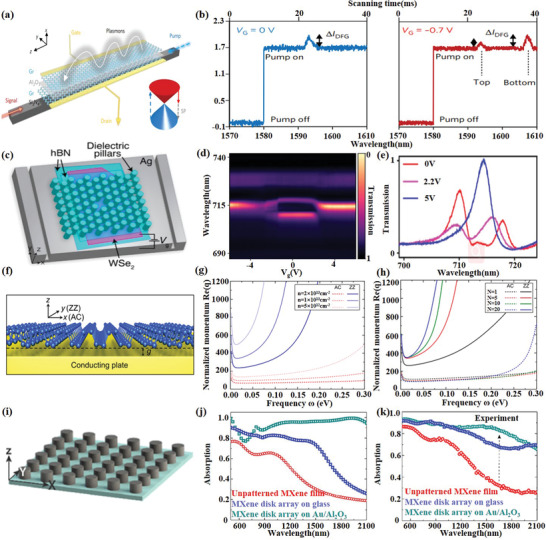
2D material‐based plasmonic devices. a) Schematic of the dual‐layer graphene hybrid for difference frequency generation (DFG). b) Measured DFG‐based signal enhancement in the optical spectra. Reproduced with permission.^[^ ^8^ ^]^ Copyright 2018, Springer Nature. c) A schematic of the plasmonic crystal cavity between two trenches etched in the silver that serve as in‐coupling and out‐coupling structures for surface plasmon polaritons (SPPs). d) Electrically tunable cavity transmission through a monolayer WSe_2_ as a function of gate voltage. e) Transmission spectra for three different gate voltages when the plasmonic crystal cavity is resonant with the exciton absorption in WSe_2_. Reproduced with permission.^[^ ^158^ ^]^ Copyright 2019, American Chemical Society. f) Geometrical configuration supporting acoustic plasmons based on BP. g) Effect of electron density on plasmon dispersion. h) The effect of the number of layers on plasmon dispersion. Reproduced with permission.^[^ ^159^ ^]^ Copyright 2018, American Chemical Society. i) Schematic of the MXene disk array. j) Simulated and k) Measured absorption spectra comparison of unpatterned MXene film, MXene 22 disk array on glass and MXene disk array on Au/alumina. Reproduced with permission.^[^ ^164^ ^]^ Copyright 2018, American Chemical Society.

Because of its simultaneously high carrier mobility and high conductivity, graphene has also emerged to be a very promising candidate for terahertz to mid‐infrared plasmonic generators applications. However, it have only been observed at mid‐IR and longer wavelengths (THz).^[^ ^149^ ^]^ In single‐layer graphene (SLG) and plasmonic resonances possess relatively low oscillator strengths because of the low attainable carrier densities. To address this issue, Constant et al.^[^ ^150^ ^]^ reported a method of all‐optical generation of graphene plasmons, which can be excited over a large frequency range with a defined wavevector and direction by carefully matching the energy and momentum using difference frequency mixing. And Rodrigo et al.^[^ ^151^ ^]^ found that the carriers density of multilayer graphene is larger than monolayer, resulting in a higher frequency resonances, stronger plasmon intensity, and wider tuning ranges. Furthermore, Brar et al.^[^ ^152^ ^]^ demonstrated highly confined tunable midinfrared plasmonic in graphene nanoresonators and Fang et al.^[^ ^153^ ^]^ demonstrated a wide range of plasmon tunability in the infrared by controlling the Fermi level of graphene (through a top‐gated ion‐gel). Recent reports, Yao et al.^[^ ^8^ ^]^ presented a two‐layer graphene heterostructure (**Figure** **10**a) that generates and controls terahertz plasmons with terahertz gate‐tunability over an octave, from 4.7 to 9.4 THz in a single‐layer graphene through a counter‐pumped all‐optical difference frequency process. It is the first time to realize the all‐optical generation and control of plasmon in the communication band. Noteworthy, the plasmonic dissipation in graphene is considerable.^[^ ^154^ ^]^ To reduce the dissipation of graphene plasmon, Woessner et al.^[^ ^155^ ^]^ encapsulated high quality graphene between two sheets of hexagonal boron nitride (hBN) to form the hBN/graphene/hBN vdWs heterostructure and investigated the propagation of plasma. The results showed that such a structure made the scattering of impurities have little effect on the damping of the plasma, significantly reduced the damping and improved the field confinement of the graphene plasma. Afterward, Ni et al.^[^ ^156^ ^]^ used the similar heterostructure to activate infrared plasmons with femtosecond optical pulses and the ultrafast dynamics of plasmons in encapsulated graphene revealed by means of nano‐IR pump–probe spectroscopy. Based on the properties and related researches of graphene plasmonic, there has been a strong focus on developing new graphene‐based plasmonic devices, ranging from tight‐field‐enhanced modulators, detectors, lasers, polarizers, and perfect absorbers to biosensors.

So far, plasmonic devices based on TMDs are rarely reported. Here, we give a brief introduction to existing reports. In 2015, Wang et al.^[^ ^157^ ^]^ successfully demonstrated plasmon resonances of MoS_2_ nanoflakes with high doping level from near UV to visible by the electrochemical intercalation/deintercalation of Li^+^ ions on MoS_2_. Recently, Dibos et al.^[^ ^158^ ^]^ reported a new electroplasmonic switcher by composing with a hBN‐WSe_2_ monolayer on top of a single‐crystalline silver substrate to enhance the coupling between excitons and plasmas, as shown in Figure 10c–e. The Fermi level of the single layer TMDs is adjusted by the gate voltage to control the coupling strength between the excitons and the plasma, thereby achieving an adjustable output of the plasma.

Compared to graphene and TMDs, BP can be used as an alternative material to support long‐life plasmon excitons with extensive tunability, robustness to surface scattering. And even more interestingly, BP is studied as an anisotropic plasmonic device because it can provide polarization‐dependent anisotropic plasma response due to their strong in‐plane anisotropy. Recent research on BP‐based plasmas is about the acoustic plasmons supported in monolayer and multilayer BP placed above conductive plates.^[^ ^159^ ^]^ This work proposes a practical high‐efficiency design for acoustic plasmon resonators, as shown in Figure 10f–h.

The metallic properties of 2D layered MXenes offer an excellent platform for light–carrier interactions at their surface. Several demonstrations have been realized to utilize the excellent plasmonic properties of MXenes. Mauchamp et al.^[^ ^160^ ^]^ found the bulk plasmon and surface plasmon behavior reversely. The ratio of SP significantly increases as the MXene thickness decrease and dominate the screening process up to 45 nm thick flakes, due to the combination of efficient free‐electron dynamics, Begrenzungs effect, and reduced interband damping. MXene Ti_3_C_2_T*_x_* nanosheets can be mixed with noble metals like Ag, Au, and Pd to achieve surface‐enhanced Raman spectroscopy (SERS) with an enhancement factor of 10^5^ for methylene blue dye.^[^ ^161^ ^]^ Furthermore, spray‐coated MXene substrates without noble metals were fabricated and used to detect rhodamine 6G, methylene blue, crystal violet, and acid blue, with enhancement factors reaching 10^6^.^[^ ^162^ ^]^ A typical Kretschmann configuration^[^ ^163^ ^]^ prism‐coupled surface plasmon resonance (SPR) sensor has been proposed as an efficient optical sensor for the biological or chemical analyte. Metasurface of Ti_3_C_2_T*_x_* nanodisk arrays was fabricated and demonstrated strong localized surface plasmon resonance at near‐infrared frequencies, plus the interband transitions in the visible regimes. This metasurface exhibits a high‐efficiency absorption (≈90%) over a broadband wavelength window of 1.55 µm, that may find applications in harvesting energy from light, biomedical imaging, and sensing.^[^ ^164^, ^165^, ^166^ ^]^

### 4.7 Sensors

In 2D material‐based optical devices, sensors are one of the most important branches, especially in gas sensing. Compared with electrochemical sensors, conventional optical sensors have achieved a breakthrough in detection sensitivity and response speed. In recent years, great progress has been made in sensor structure and devices. Various sensor structures had been proposed to realize physical,^[^ ^167^, ^168^, ^169^, ^170^ ^]^ gas,^[^ ^171^, ^172^, ^173^ ^]^ chemical, and biochemical^[^ ^174^, ^175^, ^176^, ^177^, ^178^ ^]^ sensing. The combination of optical sensors and 2D materials further makes it possible to realize selective, single‐molecular‐sensitive photobiochemical sensors,^[^ ^178^ ^]^ because 2D materials are both chemically sensitive media and optical response enhancers in sensing applications. In this section, we present some of the latest 2D material‐based sensors.

Graphene combined with optical fiber sensing technology is a new way of sensing. Most of these sensors make use of the interaction between graphene and optical fiber evanescent wave. By integrating graphene into different optical fiber structures, such as microfiber, Mach–Zehnder interferometer, D‐shaped fiber, photonic crystal fiber, and so on, it can realize various sensor designs and various parameter detections including physical quantity,^[^ ^167^ ^]^ gas,^[^ ^171^ ^]^ and biochemical sensors.^[^ ^174^ ^]^ Moreover, benefiting from its atomic thickness with ultrahigh conductivity, graphene can also realize remarkable functionalities, such as wearable sensors for smart systems^[^ ^179^ ^]^ and switching‐sensing devices for high‐precision measurements with thermal compensation.

**Figure 11:**
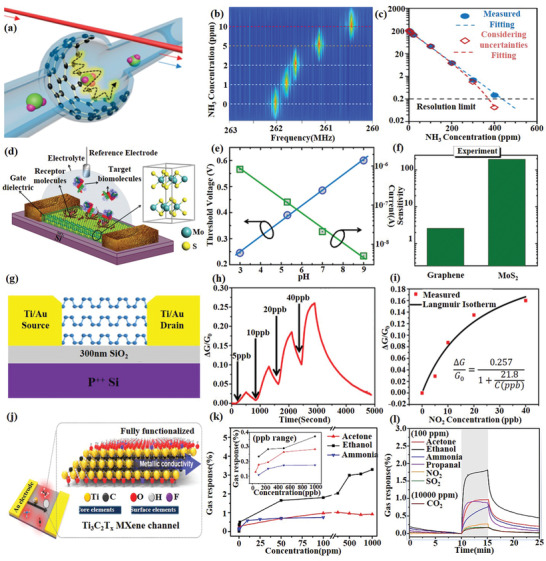
2D material‐based sensors. a) the schematic design of the graphene enhanced Brillouin microresonator. b) Colored map of the beat note spectra varies with the NH_3_ gas concentration. c) Sensitivity as a function of gas concentration. Reproduced with permission.^[^ ^171^ ^]^ Copyright 2017, American Chemical Society. d) Schematic diagram of MoS_2_‐based FET biosensor. e) Change in threshold voltage and current of the MoS_2_ FET for a wide range of pH (3–9). f) Comparison of sensitivity of graphene and MoS_2_‐based FET biosensors. Reproduced with permission.^[^ ^183^ ^]^ Copyright 2014, American Chemical Society. g) Schematic diagram of BP‐based FET gas sensor. h) Relative conductance change versus time in seconds for a multilayer BP sensor showing a sensitivity to NO_2_ concentrations (5–40 ppb). i) Relative conductance change versus NO_2_ concentration applied to the BP FET. Reproduced with permission.^[^ ^173^ ^]^ Copyright 2017, American Chemical Society. j) Schematic illustration of Ti_3_C_2_T*_x_* films and their structural and surface characterizations. k) Maximal resistance change in a wide range of diluted target gases (0.05–1000 ppm). l) Resistance variation upon exposure to 100 ppm of acetone, ethanol, ammonia, propanal, NO_2_, SO_2_, and 10 000 ppm of CO_2_ at room temperature (25 °C). Reproduced with permission.^[^ ^198^ ^]^ Copyright 2018, American Chemical Society.

In addition to the realization of more parameter sensing, further improving the sensing sensitivity through innovations in sensing structures and sensing mechanisms is the challenge that needs to be faced at present. In typical reports, Rodrigo et al.^[^ ^180^ ^]^ demonstrated a high‐sensitivity plasmonic biosensor for chemically specific label‐free detection of different protein molecules by electrically tuning the Fermi level of graphene to change the resonant frequency of the plasmons. Sun et al.^[^ ^181^ ^]^ verified the advantages of suspended graphene in gas sensing. Compared with traditional graphene coating on the surface of optical structures, there is a larger contact area between suspended graphene and gas molecules. The introduction of an electric field on the surface of graphene accelerates the adsorption process of the molecule in a low‐concentration CO_2_ environment, greatly improving the sensitivity to detect the individual physisorption while greatly reducing the response time. Yao et al.^[^ ^171^ ^]^ demonstrated a micro‐optical fiber integrated whispering gallery mode (WGM) bottle‐shaped cavity gas sensor, expanded the “electron–photon” interaction in traditional graphene optical devices to the “electron–phonon–photon” interaction process, and implemented the new promotion in the sensing performance by using a new sensing mechanism of interaction in the optomechanical microresonator. **Figure** **11**a–c shows the structural design and sensing performance, achieving ultrahigh sensitivity (1 ppb) for NH_3_ gas detection, comparable to world‐class sensing indicators.

In addition to graphene, TMD‐based photoelectronic devices exhibited high sensitivity in gas sensors and sensing platforms of DNA, proteins, and biomolecules.^[^ ^175^ ^]^ TMD‐based gas sensors achieve sensing of gas through surface–gas molecular interactions and changes in conductivity due to charge‐transfer processes when molecules are adsorbed to the surface. As early as 2013, Late et al.^[^ ^172^ ^]^ reported the gas sensing performance of different thicknesses of MoS_2_ transistors under different gate bias, light irradiation for NO_2_, NH_3_, and humidity. The results show that transistors with fewer layers of MoS_2_ exhibit better sensitivity, recovery, and ability to be manipulated by gate bias and green light compared to single‐layer ones. Conversely, Zhao et al.^[^ ^168^ ^]^ implemented an integrated humidity sensing array using a large‐area, uniform monolayer of MoS_2_. The resistance of MoS_2_ FET greatly increased in an exponential manner with RH varying from 0% to 35% and the sensitivity can be tuned by applied gate voltages and the highest value more than 10^4^. For the same use of a monolayer of MoS_2_, Pham et al.^[^ ^182^ ^]^ designed an Au/MoS_2_/Au optoelectronic gas sensor. Using red light illumination, whose photon energy matches the MoS_2_ direct bandgap to induce photocurrent, significantly enhances the device sensitivity up to 0.1 ppb of NO_2_ gas. Besides MoS_2_, other semiconductive 2D TMDs, such as MoSe_2_ and WS_2_, have also been applied for gas sensors with great performance.

Furthermore, TMD‐based biochemical sensors can be applied as label‐free detections of proteins, DNA, RNA, and small biomolecules. Using the similar structure and the same material of ref. [182], Sarkar et al.^[^ ^183^ ^]^ demonstrated FET biosensors (Figure 11d), which provides sensitivity (defined as the slope of the photocurrent change multiplied by 100) of 713 for a pH change by 1 unit in pH range (3–9) (Figure 11e) and sensitivity of 196 even at 100 × 10^−15^ m concentration for specific protein sensing. It is 74 times greater than the graphene‐based sensitivity (Figure 11f). A year later, a new platform for DNA detection was developed based on optical absorption of layered MoS_2_ and the discrimination abilities of layered MoS_2_.^[^ ^184^ ^]^ In addition, a photoluminescence (PL) sensor based on 2D TMDs was also established,^[^ ^185^ ^]^ because 2D TMD nanoparticles have strong fluorescence quenching ability and good recognition ability of biomolecules.

Black phosphorus is a layered material that is sensitive to the surrounding atmosphere. This is generally considered as a disadvantage, especially when compared to more stable layered materials, such as graphene or TMDs. But this sensitivity becomes an advantage for sensing applications. In recent years, many highly sensitive BP‐based sensors have been reported. For improving the air stability, Li et al.^[^ ^176^ ^]^ and Miao et al.^[^ ^169^ ^]^ encapsulated BP in an ionophore and a 6 nm thick package layer of Al_2_O_3_, respectively, to ensure the long‐term air stability of BP, achieving highly selective multichannel ion detection and humidity sensing with a slightly reduced sensing sensitivity. For further improving the sensitivity, a multilayer BP‐based FET^[^ ^173^ ^]^ (Figure 11g) and a suspended BP‐based FET^[^ ^186^ ^]^ were demonstrated for NO_2_ gas sensor and Hg^2+^ detection with sensitivity of 5 ppb (Figure 11h,i) and 0.1 ppb, respectively. At last, it is worth mentioning that the chemical sensing performance of BP is about 20 times higher than that of MoS_2_ and graphene during the research process of Cho et al.^[^ ^187^ ^]^ It is anticipated that this unique sensing performance of the BP will make it one of the most important materials that lead the gas sensing research fields in the near future.

Stimuli such as pressures, strains, photon irradiations, electrochemical reactions, ionic/gas adsorptions, humidity, pH values, and temperatures that alter the electrical properties (e.g., resistance, conductivity, impedance, reactance) of MXene based devices can be used to quantitatively manifest themselves. External pressure can decrease the interlayer of multilayered MXenes, in turn, reduce the internal resistance. This property can also be used to prepare a highly sensitive (gauge factor ≈180.1), fast response (<30 ms), and reversible MXene‐based piezoresistive sensor to detect human being's subtle bending‐release activities.^[^ ^188^, ^189^, ^190^ ^]^ Combined with poly(diallyldimethylammonium chloride) (PDAC), recoverable MXene‐based strain sensors with a small bending radius (2.5 mm) and large stretching limit (40%) have fabricated for detecting human motions and topographical scanning.^[^ ^191^ ^]^ Due to the low impedance of MXene, biocompatible Ti_3_C_2_T*_x_* neural electrodes exhibit higher sensitivity and resolution compared to gold electrodes.^[^ ^192^ ^]^ The active surface terminations and multilayer structures of MXenes can be utilized for the detection of various gas and biological molecules, e.g., H_2_O_2_, H_2_O, CO_2_, NH_3_, phenol, dopamine, acetaminophen, urea, etc.^[^ ^193^, ^194^, ^195^, ^196^, ^197^, ^198^, ^199^, ^200^ ^]^ Typically, the capability of charge carrier transport in MXene will be hindered when a gas molecule was adsorbed. An MXene‐based sensor has significantly pushed the detecting limit of H_2_O_2_ to 0.7 × 10^−9^ m with a response time of ≈10 s in a cathodic potential window.^[^ ^193^ ^]^

In addition to pure 2D materials, van der Waals materials including graphene‐TMD and graphene‐BP show high sensitivity to gas and biomolecular sensing. More detailed, graphene‐TMD heterostructures can be used to detect the concentration of NO_2_ with detection sensitivity of 1.2 ppm.^[^ ^201^ ^]^ And graphene‐BP heterostructure can be used in anisotropic SPR biosensors with detection sensitivity nearly 4.5 times that of single‐layer graphene‐based SPR sensors.^[^ ^202^ ^]^ In this structure, monolayer graphene not only provides binding sites for aromatic biomolecules via π–π stacking, but can also efficiently prevent BP layer degradation.

## 5 Discussions: Challenges and Opportunities

In the past decade, research on graphenes, TMDs, BP, MXenes, and other novel 2D materials has flourished in investigation community of functional information devices. So far, the 2D material‐based optoelectronic devices (including ultrafast lasers, frequency conversion devices, modulators, photodetectors, plasmonic generators and sensors) have made great progress in almost all aspects, ranging from theoretical design, material preparation, integration technique, to device configurations. All these contributions indicate the great potential of 2D materials in optoelectronic devices. Here, we summarize the properties of current 2D materials and their specific applications, as shown in **Table** **1**. However, most research to date has focused on material properties and conceptual devices. We still have great challenges in terms of practical application, and these challenges may in turn present great opportunities. Herein, based on the current development situation, we give our subjective outlook on the development trend and some of the important directions, which is hoped to shed light on the future research:

1. Optoelectronic devices with materials other than graphene. So far, most research has focused on graphene, but the unique band structure and spectral response of TMDs and black phosphorus leave a lot of room for further development.
2. Design and development of new van der Waals heterostructure. According to the specific application requirements, functional optoelectronic devices based on van der Waals heterostructure are designed, such as ultrafast lasers,^[^ ^203^ ^]^ high‐speed modulators,^[^ ^122^ ^]^ ultrasensitive sensors,^[^ ^204^ ^]^ ultrahigh responsivity photodetectors,^[^ ^205^ ^]^ and ultralow damping plasmonic.^[^ ^155^ ^]^
3. Exploration of optoelectronics applications of new metamaterials, such as perovskite^[^ ^206^, ^207^, ^208^, ^209^ ^]^ and topological insulator.^[^ ^210^, ^211^ ^]^
4. Development of new 2D material preparation process, for instance, functional inks and printing of 2D materials.^[^ ^212^ ^]^
5. Exploration of new optical mechanisms and configurations for 2D material‐based optoelectronic devices, such as phonon laser^[^ ^213^ ^]^ and exceptional points (EPs).^[^ ^214^ ^]^
6. Development of an artificial intelligence brain‐like devices based on 2D materials, combined with the current research trend of artificial intelligence.
7. Due to recent advances in the manufacturing process of 2D materials, various kinds of 2D materials can be relatively easily and economically embedded into PICs, thus achieving highly integrated, multifunctional optical devices.^[^ ^11^ ^]^
8. Modification is also a very important aspect in the development of 2D materials. By means of doping, chemical modification, electrostatic control, and alloy, the shortcomings of materials can be avoided and their advantages can be given play.
9. Realization of the wide application of 2D material optoelectronic devices. So far, a large number of studies are still in the theoretical stage. For practical applications, there are still challenges, opportunities, and huge development potential.

**Table 1:** Basic optoelectronic properties of 2D materials and their corresponding applications

Material	Optoelectronic properties	Applications
Graphene	Operating wavelength: ultraviolet to radiowaves. 2.3% of the vertically incident light absorption. Tunable bandgaps.	Broadband ultrafast lasers.^[^ ^7^, ^57^, ^58^, ^59^, ^60^, ^61^ ^]^ Light emitters.^[^ ^81^, ^82^, ^83^, ^84^, ^85^, ^86^ ^]^
	Strong third order nonlinearity.	THG,^[^ ^102^ ^]^ HHG,^[^ ^107^ ^]^ FWM,^[^ ^102^ ^]^ Optical frequency comb.^[^ ^9^ ^]^
	Bandgap:0 eV. Ultrafast carrier relaxation time. The strong light–material interaction.	Broadband modulators and photodetectors.^[^ ^112^, ^113^, ^114^, ^116^, ^117^, ^118^, ^119^, ^120^, ^121^, ^122^, ^123^, ^124^, ^125^, ^126^, ^131^, ^132^, ^133^, ^134^, ^135^, ^136^ ^]^
	High carrier mobility and high conductivity.	Plasmonic generators.^[^ ^8^, ^149^, ^150^, ^151^, ^152^, ^153^, ^155^, ^156^ ^]^
	Strong molecular adsorption capacity and light response enhancement.	Gas and biochemical sensors.^[^ ^167^, ^171^, ^174^, ^179^, ^180^, ^181^ ^]^
TMDs	Operating wavelength: visible light. The absorption of vertically incident light is up to 20%. Tunable bandgaps.	Visible to NIR ultrafast lasers.^[^ ^65^, ^66^, ^67^, ^68^, ^69^, ^70^ ^]^ Light emitters.^[^ ^87^ ^]^
	Strong second and third‐order nonlinearity related to the number of layers.	SHG,^[^ ^100^, ^101^ ^]^ THG,^[^ ^103^ ^]^ HHG,^[^ ^108^ ^]^ FWM.^[^ ^104^ ^]^
	Bandgap: 1–2.5 eV. Large carrier mobility, high‐current ON/OFF ratio and strong photoluminescence.	Visible to NIR photodetectors.^[^ ^137^, ^138^, ^139^ ^]^
	Strong fluorescence quenching ability and good recognition ability of biomolecules.	Label free detection. Gas and biochemical sensors.^[^ ^168^, ^172^, ^175^, ^182^, ^183^, ^184^ ^]^
BP	Operating wavelength: visible light to mid infrared. Strong light–material interaction. Tunable bandgaps.	Visible to MIR ultrafast lasers.^[^ ^71^, ^72^, ^73^ ^]^ Light emitters.^[^ ^88^, ^89^, ^90^ ^]^
	Strong third order nonlinearity.	THG,^[^ ^105^ ^]^ HHG,^[^ ^109^ ^]^ FWM.^[^ ^27^ ^]^
	Bandgap:0.3–2 eV. Strong field‐effect tuning.	NIR modulators and photodetectors.^[^ ^111^, ^127^, ^128^, ^140^, ^141^, ^142^ ^]^
	Strong in‐plane anisotropy.	Anisotropic plasma.^[^ ^159^ ^]^
	Sensitive to the surrounding atmosphere.	Gas sensors.^[^ ^169^, ^173^, ^176^, ^186^ ^]^
MXenes	Operating wavelength: ultraviolet to radiowaves. Optical transmission ≈1% nm^−1^. Tunable bandgaps.	Ultrafast lasers.^[^ ^40^, ^74^, ^75^, ^76^, ^77^, ^78^, ^79^ ^]^ Light emitters.^[^ ^91^, ^92^, ^93^, ^94^, ^95^, ^96^, ^97^, ^98^ ^]^
	Broadband and strong third‐order nonlinearity. Efficient photon–phonon conversion.	FWM^[^ ^106^ ^]^
	High conductivity. Tunable workfunction. Bandgap: 0–2 eV.	Modulators.^[^ ^40^, ^41^, ^129^, ^130^ ^]^
	Large mechanical moduli. 100% spin purity half‐metallic.	Plasmonic generators.^[^ ^160^, ^161^, ^162^, ^163^ ^]^
	Easy surface decoration.	Sensors^[^ ^188^, ^189^, ^190^, ^191^, ^192^, ^193^ ^]^

## Conflict of Interest

The authors declare no conflict of interest.
